# Complete mitochondrial genome of the hybrid flounder *Platichthys stellatus* (♀) × *Verasper variegatus* (♂)

**DOI:** 10.1080/23802359.2026.2664911

**Published:** 2026-07-04

**Authors:** Hyejin Kim, HoSeong Lee, Yong Hwi Kim, Bong Han Yun, Ho-Seop Han, In Gug Baek, In-Chul Bang

**Affiliations:** ^a^ Department of Biology, Soonchunhyang University, Asan, Republic of Korea; ^b^ Bio-laboratory, BioTNS Co., Ltd, Daejeon, Republic of Korea; ^c^ Institute of Korea Eco-Network, Daejeon, Republic of Korea

**Keywords:** Mitogenome, phylogenetic analysis, hybrid flatfish, aquaculture, commercial species

## Abstract

The starry flounder (*Platichthys stellatus*) and the spotted halibut (*Verasper variegatus*) are economically important flatfish species in Korea. The hybrid *P. stellatus* (♀) × *V. variegatus* (♂) has attracted attention in aquaculture. In this study, we report the first complete mitochondrial genome sequence of this hybrid flatfish, obtained using next-generation sequencing. The mitogenome is 16,874 bp in length and comprises 13 protein-coding genes, 22 tRNAs, and two rRNAs. Phylogenetic analysis revealed that the hybrid clusters with its maternal species, *P. stellatus*, confirming strict maternal inheritance of mitochondrial DNA. These findings provide genomic resources for phylogenetics and hybrid flatfish breeding.

## 1. Introduction

The starry flounder (*Platichthys stellatus* Pallas, 1787) and the spotted halibut (*Verasper variegatus* Temminck & Schlege, 1846) belong to the family Pleuronectidae of the order Pleuronectiformes. *P. stellatus* is a cold-water flatfish with high disease resistance and strong tolerance to salinity fluctuations. It primarily inhabits coastal areas at depths of up to 150 m but is also found in freshwater and brackish environments (Lim et al. 2013). This species has recently attracted attention as a promising aquaculture species in Korea due to its high market value and consumer demand (Park et al. 2016). *V. variegatus* occurs along the East Asian coast, reaching lengths of approximately 60 cm (Wada et al. 2004). It is considered a nutritious and palatable species; however, the total catch has been steadily declining, necessitating artificial breeding to secure fishery resources (Nemoto et al. 1999; Wada et al. 2004; Shimamura 2007; Wada et al. 2010). Because hybridization can enhance traits, several studies have attempted to breed hybrids among flatfish species (Riley and Thacker 1969; Lincoln 1981). For example, when hybridized with other flounder species, *P. stellatus* produces fry with rapid growth and strong environmental tolerance (Hubbs and Kuronuma 1942; Nam et al. 2008).

In this study, the complete mitochondrial genome of the hybrid *P. stellatus* (♀) × *V. variegatus* (♂) was analyzed to support further investigations into fry characteristics, hybrid differentiation, and lineage relationships; its taxonomic position was evaluated through phylogenetic analysis.

## 2. Materials

**Figure 1.**
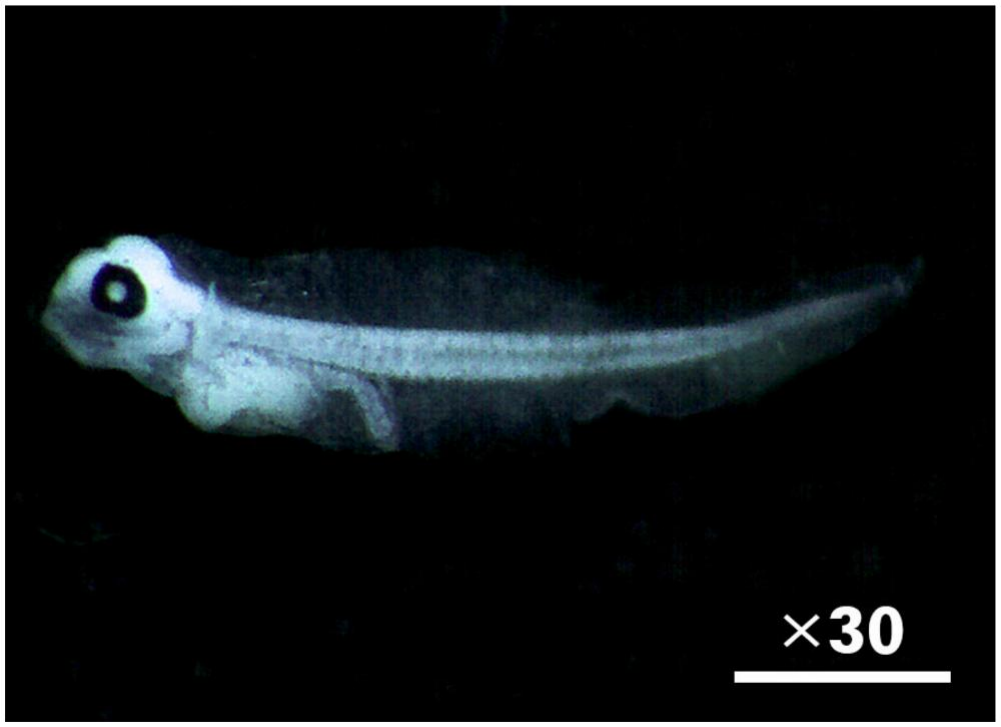
Larva of the hybrid flounder *Platichthys stellatus* (♀) × *Verasper variegatus* (♂), 4 mm in standard length (SL), collected from Taean-gun, Chungcheongnam-do, Republic of Korea, on 10 April 2023. Photographed by H. J. Kim. Scale bar = 1 mm (×30).

In March 2023, larvae of *Platichthys stellatus* (♀) × *Verasper variegatus* (♂), hatched at the Soonchunhyang University Marine Fisheries Research Institute (36.61862° N, 126.3368° E; 236 Cheonsuman, Chungcheongnam-do, Republic of Korea), were preserved in 99.9% ethanol. A specimen was deposited at laboratory 3106, Soonchunhyang University (https://gradu.sch.ac.kr/pages/sub/sub02_0102, HyeJin Kim, nsiho6@gmail.com) under the voucher number SUC25488 (Figure 1).

## 3. Methods

Genomic DNA was extracted from the whole larvae using a HiGene^™^ Genomic DNA Prep Kit (BIOFACT, Daejeon, Republic of Korea; Cat. No. GD264-060) following the manufacturer’s protocol. A DNA library was prepared using an MGIEasy DNA Library Prep Kit (MGI Tech, Shenzhen, China), and 150 bp paired-end reads were generated on the MGISEQ-2000 platform (MGI Tech, Shenzhen, China). Raw reads were trimmed using Cutadapt v4.2 (Martin 2011) and assembled in CLC Genomics Workbench v20.04 (Qiagen, Aarhus, Denmark). Circular mitochondrial contigs were confirmed in Geneious Prime (Kearse et al. 2012) and annotated with the MITOS web server (Bernt et al. 2013). The complete mitogenome sequence was deposited with GenBank (accession no. OR282488).

The assembled sequence was aligned with ClustalW (Thompson et al. 1994) in BioEdit v7.7. Model selection was performed in jModelTest v2.1.10 (Darriba et al. 2012) under the Akaike Information Criterion (AIC), identifying GTR + I + G as the best-fit model. Maximum-likelihood (ML) phylogenetic trees were constructed using PHYML v3.0 (Guindon et al. 2010) and visualized in iTOL v6 (Letunic and Bork 2007). Branch support was assessed using 1,000 bootstrap replicates. The phylogenetic framework followed Chae et al. (2023) and included three species each from *Verasper* and *Paralichthys*. Outgroups included *Acipenser fulvescens* (MT667238) and four Cynoglossidae species (*Cynoglossus roulei* MK574671; *C. semilaevis* EU366230; *C. sinicus* JQ348998; *Paraplagusia bilineata* NC023227; *P. blochii* JQ349002). The ingroup included 24 Pleuronectidae species: *Arnoglossus tenuis* (KP134337), *Cleisthenes herzensteini* (KT223828), *Glyptocephalus stelleri* (NC060723), *Hippoglossoides platessoides* (MN122825), *Hippoglossus hippoglossus* (AM749122), *H. stenolepis* (AM749126), *Kareius bicoloratus* (AP002951), *Limanda aspera* (NC028281), *Microstomus achne* (OP066370), *Platichthys stellatus* (EF424428), *Pleuronichthys cornutus* (JQ639071), *P. japonicus* (KY038655), *Pseudopleuronectes herzensteini* (ON127848), *P. yokohamae* (KT878309), *Reinhardtius hippoglossoides* (AM749130), *Verasper moseri* (EF025506), and *V. variegatus* (MK210571).

The mitochondrial genome of the hybrid flatfish (*Platichthys stellatus* ♀ × *Verasper variegatus* ♂) was assembled and visualized in Geneious Prime to confirm coverage uniformity and gene annotation completeness (Figure S1).

## 4. Results

**Figure 2.**
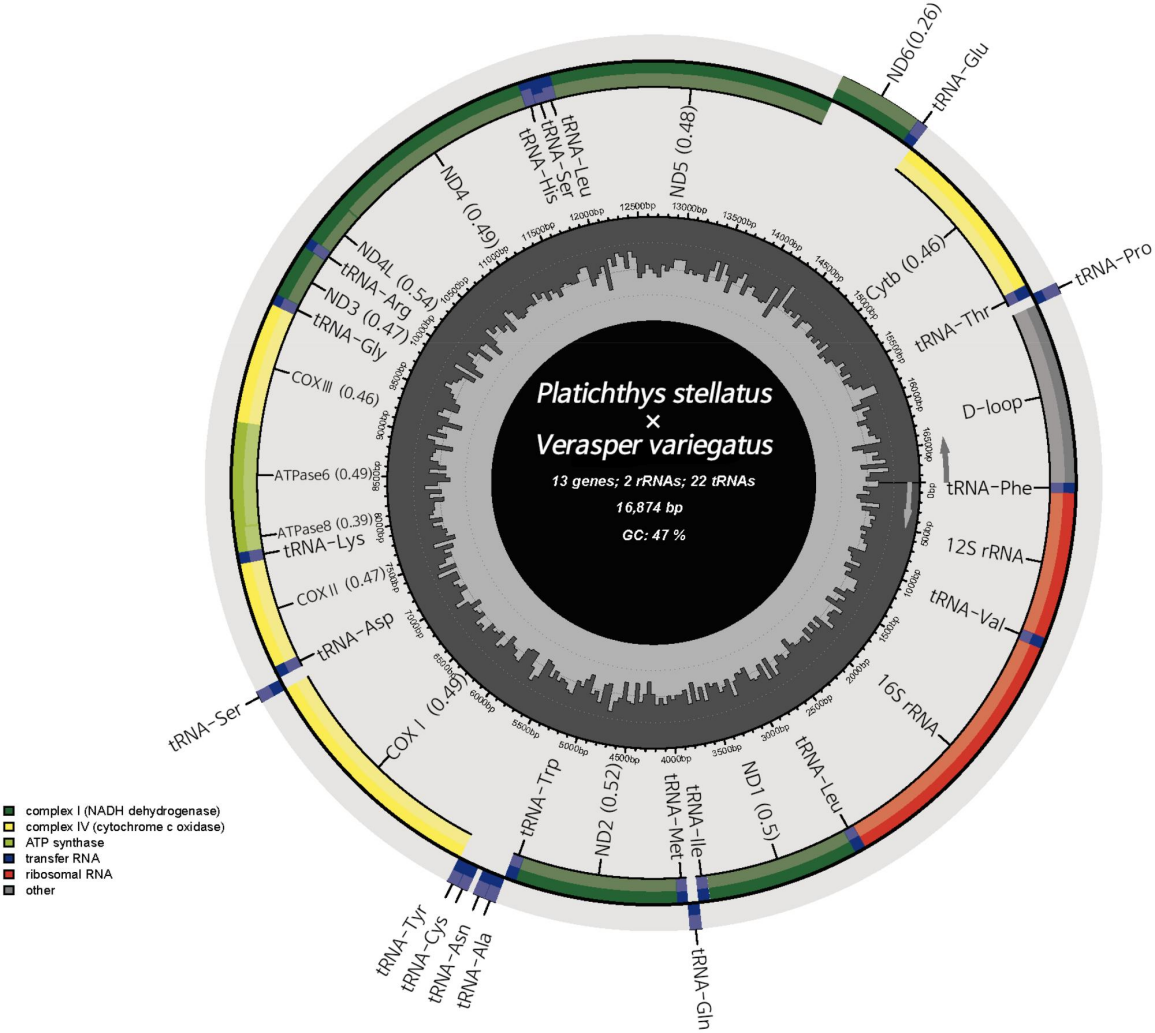
Circular map of the complete mitochondrial genome of the hybrid *Platichthys stellatus* (♀) × *Verasper variegatus* (♂) generated using the MitoFish web server v3.90. The genome is 16,874 bp in length and includes 13 protein-coding genes, 22 transfer RNAs, 2 ribosomal RNAs, and 1 control region (GenBank accession no. OR282488). The gene order and overall genomic structure are conserved among related *Pleuronectidae* species.

The complete mitochondrial genome of *Platichthys stellatus* (♀) × *Verasper variegatus* (♂) is 16,874 bp in length (GenBank accession no. OR282488) (Figure 2). It contains 13 protein-coding genes (PCGs), 22 transfer RNA (tRNA) genes, and 2 ribosomal RNA (rRNA) genes. All PCGs, except *nad6*, are encoded on the heavy strand.

To further evaluate maternal inheritance, the complete mitochondrial genome sequence of the hybrid *Platichthys stellatus* (♀) × *Verasper variegatus* (♂) was directly compared with that of the maternal species *P. stellatus* (GenBank accession no. EF424428). The mitogenome sequences were found to be identical in overall length (16,874 bp) and gene composition, including all 13 protein-coding genes, 22 tRNA genes, two rRNA genes, and the control region. Pairwise sequence comparison revealed 100% nucleotide identity between the hybrid and the maternal *P. stellatus* mitogenome across the entire mitochondrial genome, with no detectable substitutions or indels. This complete sequence concordance provides direct evidence of strict maternal inheritance of mitochondrial DNA in the hybrid individual.

The overall base composition is A 26.99%, C 29.15%, G 17.71%, and T 24.36%, yielding an A + T content of 51.35%, similar to other vertebrate mitogenomes (Saccone et al. 1999; Zhu et al. 2013). The *12S rRNA* (949 bp) is located between *tRNA*^Phe^ and *tRNA*^Val^; the *16S rRNA* (1715 bp) is between *tRNA*^Val^ and *tRNA*^Leu^.

Among the 13 PCGs, 11 genes, excluding *COX1* and *ND6*, initiate with the ATG start codon. Four PCGs terminate with a complete stop codon (TAG or TAA), whereas *ND2*, *COX2*, *ATP8*, *COX3*, *ND3*, *ND4L*, *ND4*, *ND6*, and *CYTB* end with incomplete stop codons (T or A). The combined length of all PCGs is 11,269 bp, accounting for 66.78% of the total mitogenome. The control region is 1170 bp long.

This length is comparable to the control regions reported in other Pleuronectidae species, which generally range from approximately 1100–1200 bp.

The average sequencing depth across the complete mitochondrial genome was approximately 1757×, indicating uniform and sufficient coverage for reliable genome assembly and annotation.

**Figure 3.**
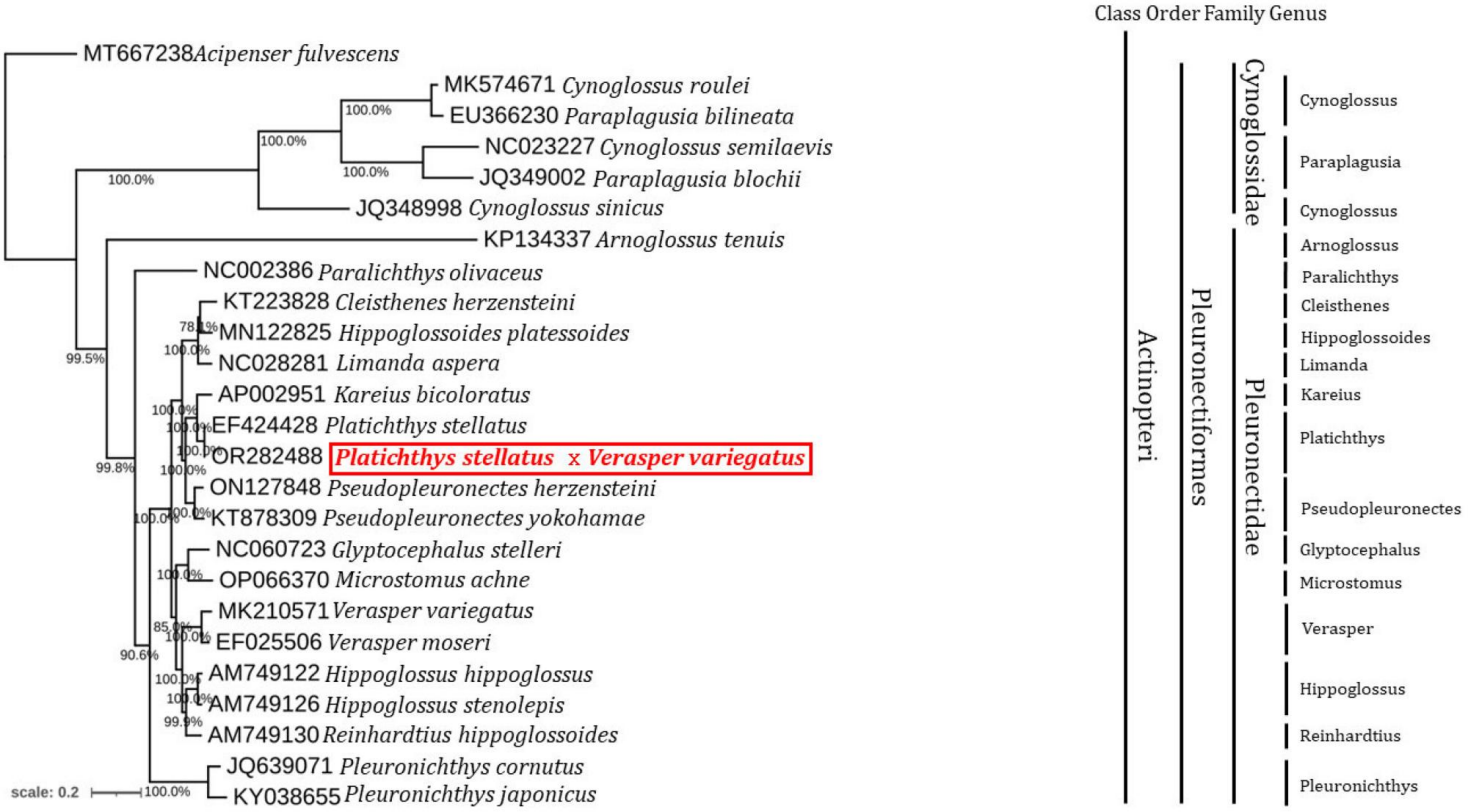
Maximum-likelihood (ML) phylogenetic tree based on 13 mitochondrial protein-coding genes (PCGs) from *Platichthys stellatus* (♀) × *Verasper variegatus* (♂) and 13 Pleuronectidae species. GenBank accession numbers precede species names. The analysis included *Acipenser fulvescens* (MT667238; Schroeter et al. 2020), *Cynoglossus roulei* (MK574671; Chen et al. 2019), *C. semilaevis* (EU366230; Kong et al. 2009), *C. sinicus* (JQ348998; Shi et al. 2015), *Paraplagusia bilineata* (NC023227; Fricke et al. 2024), *P. blochii* (JQ349002; Li et al. 2016), *Arnoglossus tenuis* (KP134337; Li et al. 2015), *Paralichthys olivaceus* (NC002386; Saitoh et al. 2000), *Cleisthenes herzensteini* (KT223828; Bo et al. 2016), *Glyptocephalus stelleri* (NC060723; Xiao et al. 2010), *Hippoglossoides platessoides* (MN122825; Margaryan et al. 2021), *H. hippoglossus* (AM749122; Mjelle et al. 2008), *H. stenolepis* (AM749126; Vinnikov et al. 2018), *Kareius bicoloratus* (AP002951; Miya et al. 2001), *Limanda aspera* (NC028281; Vinnikov et al. 2018), *Microstomus achne* (OP066370; Chae et al. 2023b), *Platichthys stellatus* (EF424428; Vinnikov et al. 2018), *Pleuronichthys cornutus* (JQ639071; Shi et al. 2015), *P. japonicus* (KY038655; Song et al. 2017), *Pseudopleuronectes herzensteini* (ON127848; Chae et al. 2023a), *P. yokohamae* (KT878309; Liu et al. 2017), *Reinhardtius hippoglossoides* (AM749130; Mjelle et al. 2008), *Verasper moseri* (EF025506; He et al. 2008), and *V. variegatus* (MK210571; Lim et al. 2019). The hybrid (*P. stellatus* ♀ × *V. variegatus* ♂) clustered with the maternal species *P. stellatus*, forming a sister relationship with *Kareius bicoloratus* within the genus *Platichthys.*

Phylogenetic analysis based on the complete mitogenome indicated that *P. stellatus* (♀) × *V. variegatus* (♂) is most closely related to the maternal species *P. stellatus*, forming a sister clade with *P. bicoloratus* within the genus *Platichthys* (Figure 3).

## 5. Discussion and conclusions

This is the first report of the complete mitogenome sequence of *Platichthys stellatus* (♀) × *Verasper variegatus* (♂) larvae using next-generation sequencing technology. The mitogenome composition aligns with that of a typical vertebrate (Pereira 2000).

In phylogenetic analyses, the hybrid flounder clustered within the same clade as its maternal species, *P. stellatus*, consistent with maternal inheritance of mitochondrial DNA. Similar results have been reported for other hybrid taxa (Debes et al. 2013; Hou and Liu 2018; Li et al. 2018), implying that the mitochondrial genome of hybrids is predominantly influenced by the maternal lineage. This finding is particularly meaningful because *P. stellatus*, the maternal species, has several advantageous physiological traits such as epidermal mucus containing antibacterial proteins effective against *Staphylococcus aureus* and methicillin-resistant *S. aureus* (Kasai 2010). In addition, it exhibits excellent osmoregulatory capacity and tolerance to photohaline and low temperatures, leading to a higher survival rate than *Paralichthys olivaceus* (Kang 2011; Ko et al. 2019; Kim et al. 2025). Therefore, the predominance of the maternal mitogenome in the hybrid *P. stellatus* (♀) × *V. variegatus* (♂) supports its potential as a commercially valuable aquaculture species with enhanced environmental adaptability. The corresponding mitochondrial genome will serve as a foundational resource for molecular phylogenetic and taxonomic studies within the genus *Platichthys*, aiding the identification of species with traits favorable for aquaculture development.

## Supplementary Material

Supplemental Material

## Data Availability

The genome sequence data that support the findings of this study are openly available in GenBank of NCBI at [https://www.ncbi.nlm.nih.gov] under the accession no. OR282488. The associated BioProject, SRA, and Bio-Sample numbers are PRJNA1010962, SRS18750687, and SAMN37204529 respectively.
