# Supplementary material for: Complete mitochondrial genome of the hybrid flounder Platichthys stellatus (♀) × Verasper variegatus (♂)
Source: Mitochondrial DNA B Resour. 2026 Jul 4;11(8):919–24. doi: 10.1080/23802359.2026.2664911 (PMC13347844; doi:10.1080/23802359.2026.2664911)

Figure S1. Mitochondrial genome coverage and gene annotation of the hybrid flatfish (*Platichthys stellatus* ♀ × *Verasper variegatus* ♂). The complete mitochondrial genome of the hybrid flatfish was mapped against the *P. stellatus* reference sequence. The upper blue histogram indicates sequencing depth across the mitochondrial genome, showing uniform coverage with an average depth of approximately 1,757×. Gene annotations are displayed below the coverage plot, including 13 protein-coding genes (*cox1–3*, *atp6*, *nad1–6*, *cytb*), two ribosomal RNA genes (*rrnS, rrnL*), 22 transfer RNA genes, and the control region. The consistent coverage and conserved gene order confirm the maternal inheritance and integrity of the mitochondrial genome in the hybrid individual.


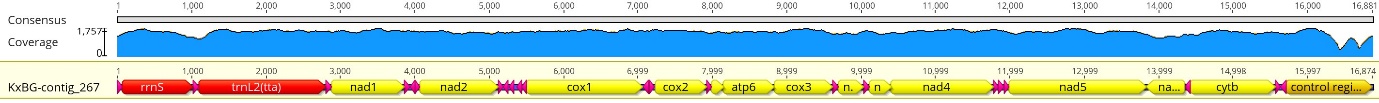

Supplement: Supplemental Material [file TMDN_A_2664911_SM3300.docx]
